# Economic Evaluation and Budget Impact Analysis of Vaccination against *Haemophilus influenzae* Type b Infection in Thailand

**DOI:** 10.3389/fpubh.2017.00289

**Published:** 2017-11-20

**Authors:** Surachai Kotirum, Charung Muangchana, Sirirat Techathawat, Piyameth Dilokthornsakul, David Bin-Chia Wu, Nathorn Chaiyakunapruk

**Affiliations:** ^1^School of Pharmacy, Monash University Malaysia, Bandar Sunway, Malaysia; ^2^Faculty of Pharmacy, Social and Administrative Pharmacy Department, Rangsit University, Muang Pathum Thani, Thailand; ^3^National Vaccine Institute (Public Organization), Ministry of Public Health, Nonthaburi, Thailand; ^4^Center of Pharmaceutical Outcomes Research (CPOR), Faculty of Pharmaceutical Sciences, Department of Pharmacy Practice, Naresuan University, Phitsanulok, Thailand; ^5^School of Pharmacy, University of Wisconsin, Madison, WI, United States; ^6^Asian Centre for Evidence Synthesis in Population, Implementation and Clinical Outcomes (PICO), Health and Well-being Cluster, Global Asia in the 21st Century (GA21) Platform, Monash University Malaysia, Bandar Sunway, Malaysia

**Keywords:** economic evaluation, budget impact analysis, *Haemophilus influenzae*, Hib, *Haemophilus* vaccines, vaccination, Thailand

## Abstract

Current study aimed to estimate clinical and economic outcomes of providing the *Haemophilus influenzae* type b (Hib) vaccination as a national vaccine immunization program in Thailand. A decision tree combined with Markov model was developed to simulate relevant costs and health outcomes covering lifetime horizon in societal and health care payer perspectives. This analysis considered children aged under 5 years old whom preventive vaccine of Hib infection are indicated. Two combined Hib vaccination schedules were considered: three-dose series (3 + 0) and three-dose series plus a booster does (3 + 1) compared with no vaccination. Budget impact analysis was also performed under Thai government perspective. The outcomes were reported as Hib-infected cases averted and incremental cost-effectiveness ratios (ICERs) in 2014 Thai baht (THB) ($) per quality-adjusted life year (QALY) gained. In base-case scenario, the model estimates that 3,960 infected cases, 59 disability cases, and 97 deaths can be prevented by national Hib vaccination program. The ICER for 3 + 0 schedule was THB 1,099 ($34) per QALY gained under societal perspective. The model was sensitive to pneumonia incidence among aged under 5 years old and direct non-medical care cost per episode of Hib pneumonia. Hib vaccination is very cost-effective in the Thai context. The budget impact analysis showed that Thai government needed to invest an additional budget of 110 ($3.4) million to implement Hib vaccination program. Policy makers should consider our findings for adopting this vaccine into national immunization program.

## Introduction

*Haemophilus influenzae* type b (Hib) is a leading cause of childhood bacterial meningitis, pneumonia, and other serious infections, associated with substantial morbidity and mortality among children under 5 years of age (1). About 3–6% among Hib cases are fatal and up to 20% of patients who survive Hib meningitis have permanent hearing loss or other long-term neurological sequelae (2). Routine Hib vaccination program led to a near eradication of Hib diseases in high-income countries (3).

Recommended by the World Health Organization (WHO), Hib vaccine has been introduced into the Expanded Program on Immunization (EPI) in some low- and middle-income countries (LMICs) with the support from the Gavi, the Vaccine Alliance (4–8). Despite Gavi support, including Hib vaccine into EPI in the LMICs was delaying far behind from all high-income countries (9). Thailand and China are the only two countries in the whole world that has not introduced Hib vaccine as part of EPI (10). This is due to uncertainties around low Hib disease burden and high vaccine price, although it has been available in Thailand since 2001 (11, 12). With substantial financial barriers to implementing EPI, economic evaluation on vaccine is essential and highly recommended to inform vaccine decision-making (6, 13). As of now, there was only one economic evaluation of universal Hib vaccination based on cost-benefit analysis (CBA) for Thailand conducted in year 2010 (14). It showed that the Hib vaccine was only cost-effective if intangible benefits were included in the model. The other factors contributing to unfavorable outcomes were vaccine unit price and Hib disease incidence.

There are a number of reasons that highlight the need for updated and more comprehensive economic evaluation of this vaccine. First, the previous economic evaluation did not capture the non-meningitis non-pneumonia infection, which is one of important consequences of Hib infection. Second, the previous economic evaluation was CBA under provider and client perspectives which was not the requirement by Thai government health decision makers that need cost-utility analysis (CUA) under societal perspective to compare Hib vaccine with other health interventions. Last, the vaccine decision-making process in Thailand has undergone major changes. Before 2003, EPI committee directly informed Thai Ministry of Public Health (MOPH) decides vaccines to be included in EPI. After 2003, vaccine incorporation into EPI needed to go through two more appraisal bodies including the National Health Security Office (NHSO) currently taking hold of national vaccine fund and the National List of Essential Medicines (NLEM). In addition, since 2011, new information has been established, e.g., reduction of vaccine acquisition cost (price), vaccine effectiveness (VE) from a recent meta-analysis, and the cost-effectiveness threshold of Thai baht (THB) 160,000 ($4,878) per QALY gained in Thailand, which is useful in guiding reimbursement of medicines using a CUA recommended in the Thailand’s Health Technology Assessment (HTA) guideline (15–17). To better understand the simultaneous impact of those factors on the health economic value of Hib vaccine, we performed a CUA of universal Hib vaccination requested by National Vaccine Institute to inform decisions in including Hib vaccine as a part of this country’s EPI using the most updated available information.

## Materials and Methods

### Overall Description

A CUA was undertaken to estimate costs and health outcomes of routine Hib vaccination (DTP-HepB-Hib: diphtheria, tetanus, pertussis, hepatitis B, and Hib infections) with three-dose primary series at 2, 4, and 6 months with or without booster dose (3 + 0 and 3 + 1 schedules) compared to “no vaccination” (DTP-HepB) among children in Thailand. Since some cases of Hib meningitis have long-term sequelae, i.e., hearing loss, epilepsy, the lifetime time horizon was used. Discount rate of 3% was used for costs and health outcomes. Results were presented incremental cost-effectiveness ratio (ICER) in terms of monetary value (THB) per quality-adjusted life year (QALY) gained. Societal perspective was used recommended by Thailand’s HTA Guideline (18).

**Figure 1:**
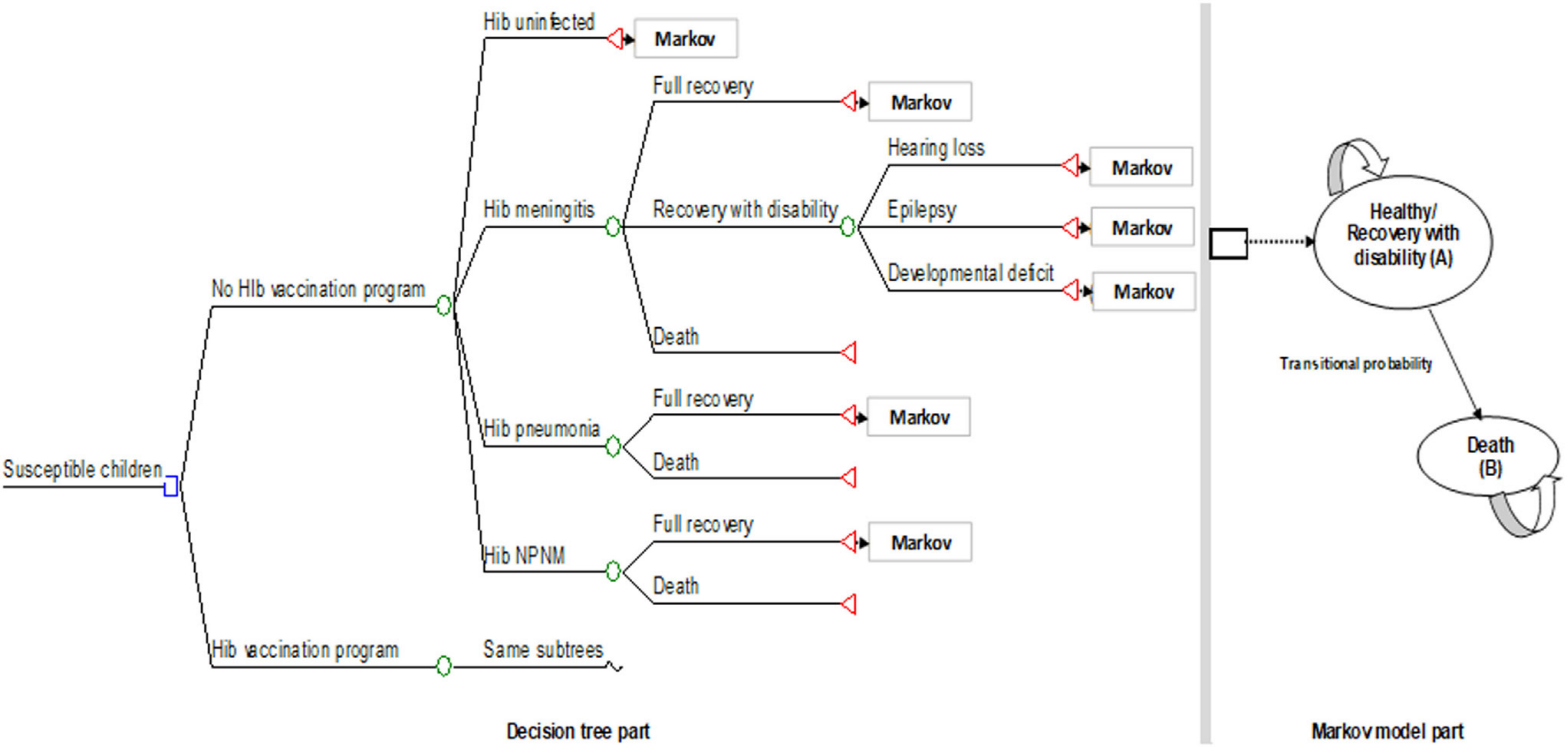
Decision tree combined with Markov model. Model used for evaluating costs and health-related outcomes of DTP-HepB-Hib vaccination national program compared to no vaccination (The structure of the vaccination program node is same to no vaccination node). NPNM, non-pneumonia non-meningitis; Hib, *Haemophilus influenzae* type b.

Decision tree combined with Markov model was constructed based on the natural history of diseases from Hib infection (Figure 1). Decision tree captures Hib infections occurred during the first 5-year of life, while Markov captures long-term outcomes throughout their life. The decision tree model captures the incidence of Hib meningitis, Hib pneumonia, Hib non-pneumonia non-meningitis (NPNM, including sepsis/bacteremia, cellulitis, and epiglottitis) during the first 5-year of life since Hib infections occur in this period of time. Hib meningitis leads to three clinical consequences including full recovery, recovery with sequelae, and death. Sequalae included hearing loss, epilepsy, and developmental deficit. The Markov model captures long-term consequences after 5-year period in decision tree model. Patients who survived from acute Hib infection were moved to Markov states which were classified into four heath statuses including (1) healthy (full recovery), (2) survived with hearing loss, (3) survived with epilepsy, and (4) survived with developmental deficit. Patients could die with different mortality rate depending on their health status after acute Hib infection. The herd effect and the vaccine adverse effects were not considered in this model. Cycle length of 1 year was used. We assumed that Hib infection could occur only once for each individual.

### Model Input Parameters

Model input parameters were obtained from retrospective electronic hospital database of NHSO and published evidence (Tables 1–3). Five types of stakeholders, including health economist, health care providers (pediatrician and pediatric infectious disease specialist), patients’ representative, third-party payers, and representatives from pharmaceutical industry have participated in two stakeholder consultation meetings in order to define study population, intervention, model structure, outcome, and comparator, assumptions used, validating data inputs, providing comments on interim findings.

**Table 1:** Epidemiological data used in the model.

Parameters	Base-case	Range (min—max)	Reference
Thai birth cohort population	782,129	–	(19)
Incidence of haemophilus influenzae type b (Hib) meningitis in <1 year old	0.000137	0.000038–0.000236	(14, 20)
Incidence of Hib meningitis in ≥1 to <5 years old^a^	0.000046	0.000038–0.000054	
Incidence of Hib pneumonia in <5 years	0.000959	0.000266–0.001652	(21)^b^
Incidence of Hib NPNM in population <5 years old	0.000025	0.000007–0.000043	(1)^c^

**Case-fatality ratio of Hib meningitis**			

Estimate based on real-world evidence of national database (base-case analysis)	0.029	0.018–0.040	NHSO^d^
Estimate from published article	0.099	0.049–0.149	(22)

**Case-fatality ratio of Hib pneumonia**			

Estimate based on real-world evidence of national database (base-case analysis)	0.019	0.012–0.026	NHSO^d^
Estimate from published article	0.020	0.010–0.030	(1)

**Case-fatality ratio of Hib NPNM**			

Estimate based on real-world evidence of national database (base-case analysis)	0.050	0.022–0.078	NHSO^d^
Estimate from published article	0.010	0.005–0.015	(1)
Probability of hearing loss after recovery from Hib meningitis	0.111	0.055–0.167	
Probability of epilepsy after recovery from Hib meningitis	0.050	0.025–0.075	(23)
Probability of developmental deficit after recovery from Hib meningitis	0.083	0.041–0.125	

**Risk ratio of mortality compared to general population**			

Hearing loss	1.075	1.010–1.140	
Epilepsy	1.005	1.000–1.010	(24)
Developmental deficit	6.165	5.160–7.170	

*NHSO, National Health Security Office; NPNM, non-pneumonia non-meningitis*.

*^a^Incidence of Hib meningitis in population ≥5 years old = 0*.

*^b^Adjustment to seven times of Hib meningitis incidence in Thai population <1 year old based on that stated reference*.

*^c^Adjustment to 5.4 times lower than Hib meningitis incidence in Thai population <1 year old based on that stated reference*.

*^d^Analysis of NHSO database using International Classification of Disease codes for identification of Hib-infected cases*.

**Table 2:** Vaccine effectiveness, coverage, wastage, and price data used in the model.

Parameters	Base-case	Range (min–max)	Source(s)
Coverage (%) of one-, two-, three-, or four-dose vaccine exposure among Thai children^a^	100, 99.7, 99.4, 97.8	–	DCD^b^

**Vaccine effectiveness (summary odds ratio) of haemophilus influenzae type b (Hib) meningitis reduction**			

One-dose exposure	0.450	0.200–0.980	
Two-, three-, and four-dose exposure	0.040	0.010–0.140	

**Vaccine effectiveness (summary odds ratio) of Hib pneumonia/NPNM reduction**			

One-dose exposure	0.410	0.240–0.700	(15)
Two-dose exposure	0.089	0.031–0.260	
Three- and four-dose exposure	0.030	0.010–0.130	
DTP-HepB-Hib acquisition cost (THB) per dose^a^	148.50	–	NVI^c^
Wastage rate (%) of single-dose vaccine formulation^a^	5.00	–	(29)
DTP-HepB acquisition cost (THB) per dose^a^	42.05	–	DMSIC^d^
Wastage rate (%) of multiple-dose vaccine formulation^a^	38.70	–	(29)

*DCD, Disease Control Department; NVI, National Vaccine Institute; DMSIC, Drug and Medical Supply Information Centre; THB, Thai baht*.

*^a^These input parameters were fixed or were not assigned a distribution for randomly selection under probabilistic sensitivity analysis performing*.

*^b^Cluster survey of DTP-HepB vaccine coverage by the DCD, Ministry of Public Health of Thailand*.

*^c^Price survey of DTP-HepB-Hib vaccine by the NVI*.

*^d^Average price from DMSIC, Ministry of Public Health of Thailand*.

**Table 3:** Cost data (Thai baht, year of costing: 2014) and utility data.

Parameters	Base-case	Range (min-max)	Source(s)
**Direct medical care costs**			
NHSO database (base-case analysis)			
Medical care cost per hospitalized haemophilus influenzae type b (Hib) meningitis episode <1 years old	36,244	34,155–38,333	NHSO
Medical care cost per hospitalized Hib meningitis episode 1 to <5 years old	21,648	19,612–23,684	
Medical care cost per hospitalized Hib pneumonia episode <1 year old	33,784	32,074–35,494	
Medical care cost per hospitalized Hib pneumonia episode <5 years old	21,320	19,617–23,023	
Medical care cost per hospitalized Hib NPNM episode <5 years old	16,236	13,612–18,860	
Previous cost estimation study [consumer price index (CPI) adjustment for all cost items]			
Medical care cost per hospitalized Hib meningitis episode <5 years old	74,687	65,639–83,735	
Medical care cost per hospitalized Hib pneumonia episode <5 years old	51,519	47,227–55,811	(30)
Medical care cost per hospitalized Hib NPNM episode <5 years old	14,646	9,888–19,404	
Previous cost estimation study (separately adjusted for labor + CPI for other cost items)			
Medical care cost per hospitalized Hib meningitis episode <5 years old	165,552	131,830–199,274	
Medical care cost per hospitalized Hib pneumonia episode ( 5 years old	105,088	92,282–117,894	(30)
Medical care cost per hospitalized Hib NPNM episode <5 years old	14,646	9,888–19,404	
Direct medical cost from disability of each age group			
Annual cost of hearing loss aged ≤14 years old	929	530–1,328	
Annual cost of hearing loss aged 15–59 years old	869	819–919	
Annual cost of hearing loss aged ≥60 years old	1,361	1,233–1,489	
Annual cost of epilepsy aged ≤14 years old	4,521	4,028–5,014	
Annual cost of epilepsy aged 15–59 years old	1,660	1,638–1,682	(24)
Annual cost of epilepsy aged ≥60 years old	1,734	1,646–1,822	
Annual cost of developmental deficit aged ≤14 years old	3,716	1,296–6,136	
Annual cost of developmental deficit aged 15–59 years old	971	896–1,046	
Annual cost of developmental deficit aged ≥60 years old	6,028	3,028–9,028	
**Direct non-medical care costs**			
Meningitis per episode	16,062	0–32,124	
Pneumonia per episode	5,886	0–11,772	
NPNM per episode	10,359	0–20,718	(24)
Hearing loss per year	900	0–1,800	
Epilepsy per year	4,656	0–9,312	
Developmental deficit per year	18,202	0–36,404	
**Utility data**			
Utility weight of meningitis health state	0.340	0.248–0.432	
Utility weight of pneumonia health state	0.590	0.513–0.667	
Utility weight of NPNM health state	0.550	0.468–0.632	(24, 33)
Utility weight of hearing loss health state	0.540	0.489–0.591	
Utility weight of epilepsy health state	0.640	0.548–0.732	
Utility weight of developmental deficit health state	0.100	0.000–0.222	
Discount rate (% per annum)	3	0–6	(18)

*NPNM, non-pneumonia non-meningitis; NHSO, National Health Security Office*.

### Epidemiological Data

The disease incidences were derived from prospective studies of Hib meningitis incidence in Thailand and a previously published economic evaluation study (14, 20, 25). While incidence of other Hib infections was not available, the incidence of Hib pneumonia was assumed as 7 times (range of 4–10 times) of Hib meningitis incidence based on WHO estimation (21). The incidence of Hib NPNM infection consisting mainly of bacteremia was estimated to be 5.4 times less than that of Hib meningitis based on global estimates (1).

Case-fatality ratios (CFRs) associated with Hib infections were derived using electronic hospital database of NHSO covering 80% of insured Thai population during January 2008 and December 2013. CFR was calculated based on patients less than 5 years of age with primary diagnosis code of Hib meningitis [International Classification of Disease (ICD)-10: G00.0], Hib pneumonia (ICD-10: J14), or sepsis due to Hib (ICD-10: A41.3)]. The CFR of Hib meningitis, pneumonia, and sepsis that assumed to represent Hib NPNM were 2.9% (1.8–4.0%), 1.9% (1.2–2.6%), and 5.0% (2.2–7.8%), respectively. In addition, CFR from international estimates were alternatively used in a scenario analysis (1, 22). The probabilities of developing hearing loss, epilepsy, and development deficit after Hib meningitis were based on estimates in previous CBA and meta-analysis studies (14, 23). The relative risks of dying among those with sequalae were based on previous CUA study (24). These relative risks were multiplied with Thai age-specific mortality to derive the likelihood of dying from all health states in the Markov model (26) (Table 1).

### VE and Coverage Rate

Vaccine effectiveness against vaccine-type Hib meningitis and invasive Hib disease (pneumonia and NPNM) was based on a systematic review and meta-analysis (Table 1) (15, 27, 28). It was assumed that the vaccination effect lasted over a 5-year period (15). Vaccination coverage rate was based on 2013 cluster survey study of DTP-HepB vaccine among Thai children conducted by Department of Disease Control of Thai MOPH (personal communication with expert in EPI committee) (Table 2).

### Costs and Outcomes

Both direct medical and direct non-medical costs were estimated. It was assumed that lost or impaired ability to work or engage in leisure activities due to morbidity would be captured in the disutility of QALY. Indirect costs were thus not included to evade double counting (18). The cost of the vaccination program included vaccine acquisition cost and wastage cost based on wastage rate of single-dosed DTP-HepB-Hib and multiple-dosed DTP-HepB vaccines from Thailand’s survey study (29). Direct medical costs for hospitalized episode of Hib infection were obtained from three cost estimation approaches in order to provide a broader possible range of cost estimates. The first approach was to derive cost estimates based on DRG using the abovementioned NHSO database (2008–2013). The adjusted relative weights (adjRW) of Hib-specific infections were multiplied with base-rate reimbursement of THB 8,200 ($250) for 1 adjRW. These costs were used in base-case analysis. The second approach was based on cost estimates obtained from a study determining cost estimates for Hib-specific infection, conducted in Thailand in year 2002 in 42 patients (30). We adjusted the estimates using medical care consumer price index (CPI) for the whole aggregated value from 2002 to 2014. The third approach was similar to the second approach where both were based on cost estimates from a study in Thailand (30). However, in the third approach, we adjusted labor cost item with the ratio of the aggregated amount for wage and salary of health care professionals of 2002 and 2014 by Thai Ministry of Health, while other cost items were adjusted using CPI (Table 1) (31). The rationale for the separated adjustment for labor cost was the observed substantial increase of health care professionals’ compensation rates in the last decade. The cost estimates from the second and third approach were used in scenario analyses. Direct non-medical costs, including transportation, meals, accommodation, facilities, caregivers’ productivity loss for hospital visits, or providing informal care, were derived from a previous study (24).

The budget impact analysis was analyzed to estimate the whole budget required and the real incremental budget for implementing of national DTP-HepB-Hib vaccination program compared to DTP-HepB in EPI among 782,129 Thai children (the number of cohort of children in year 2013). All cost values are presented in 2014 and could be converted to US dollars ($) using exchange rates THB 32.8 = $1 for international comparison (32) (Table 3).

### Utility

Utility reduction was not applied for healthy individuals in our model. For infected individuals, we applied their utility weights deriving from published studies that conducted among Thai patients in the same health states of Hib infection (Table 3) (24, 33). The utility weights were based on Health Utility Index mark 3, which they were used to analyze the cost-utility of pneumococcal vaccine under the Thai context (24).

### Base-Case Analysis

Primary outcomes were the difference of Hib infections with/without sequelae and death over 5 years, incremental costs, life year gained, QALYs gained, and ICER. For base-case analysis, we calculated the expected lifetime costs and outcomes for each program. The results are presented as ICER of DTP-HepB-Hib versus DTP-HepB as a part of EPI. An official willingness-to-pay (WTP) of the Thai Health Economic Working Group threshold (THB 160,000; $4,878 per QALY gained) for drug listing in NLEM year 2012 was used as cost-effectivenessthreshold (16, 17).

### Sensitivity Analysis

**Figure 2:**
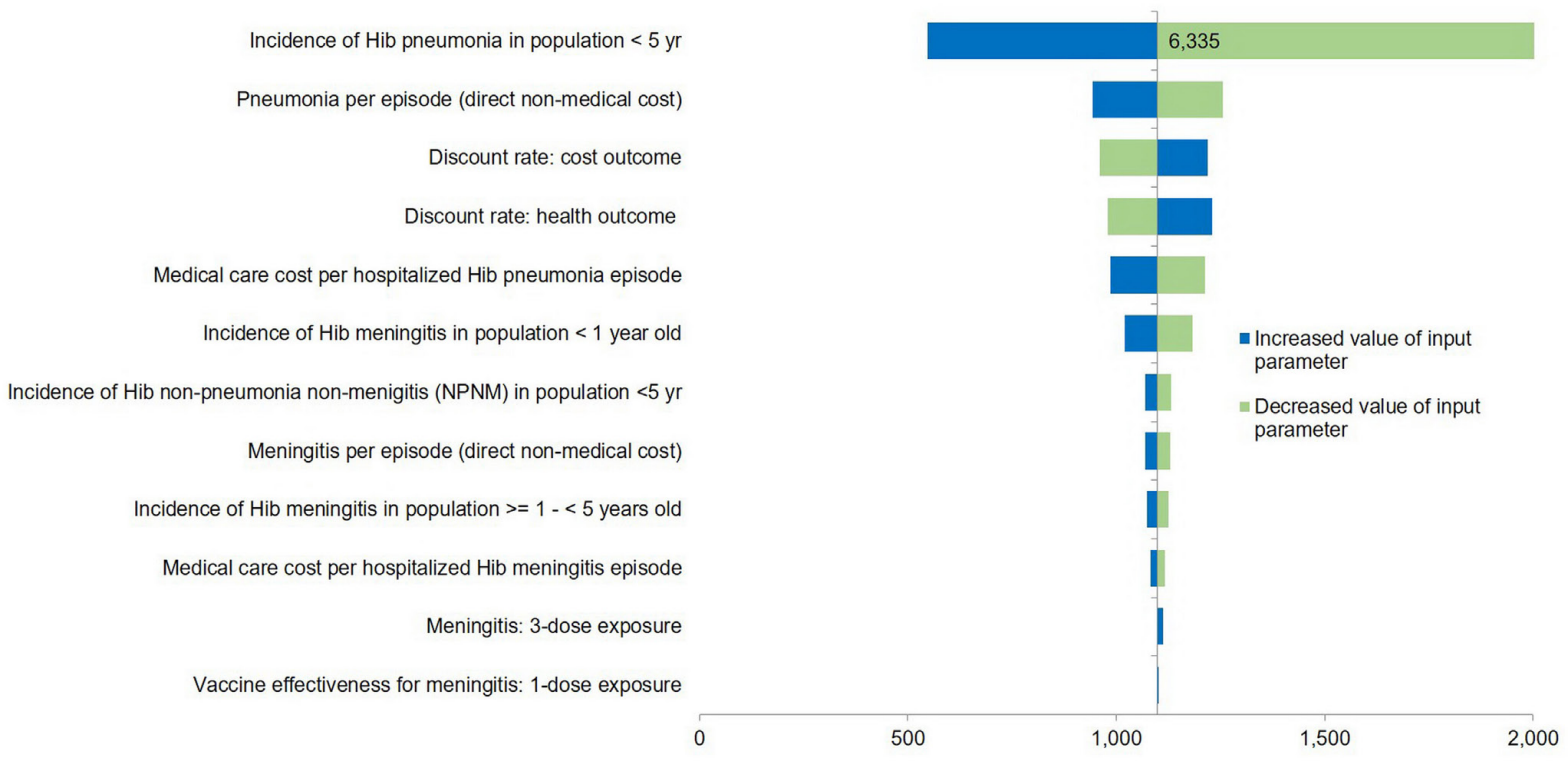
Tornado diagram of one-way sensitivity analysis (3 + 0 vaccination schedule with NHSO costing data). Tornado diagram summarized the results of one-way sensitivity analysis to evaluate the input uncertainties within plausible ranges of 95% confidence interval. The influential factors were listed descending with the variation of value. The vertical line represents the base-case incremental cost-effectiveness ratio (ICER). *X*-axis indicates the ICER. NHSO, National Health Security Office; Hib, *Haemophilus influenzae* type b.

One-way sensitivity analysis was performed to evaluate the uncertainties surrounding each input within plausible ranges of 95% CI including disease incidence, VE, costs, utilities, and discounting rate at 0 and 6% per annum and were presented using tornado diagram (Figure 2). Scenario analyses were performed when a four-dose of DPT-HB-Hib regimen (at 2, 4 and 6 months of age plus a booster dose at age between 12 and 18 months) or 3 + 1 schedule was considered, assuming the similar effectiveness between 3 + 0 and 3 + 1 dose regimens (15, 34). Scenario analyses were also performed using another two different cost estimating approaches as described previously CFR of Hib-specific infections based on published article was used in another scenario analysis. We also provide threshold analysis for vaccine acquisition cost.

**Figure 3:**
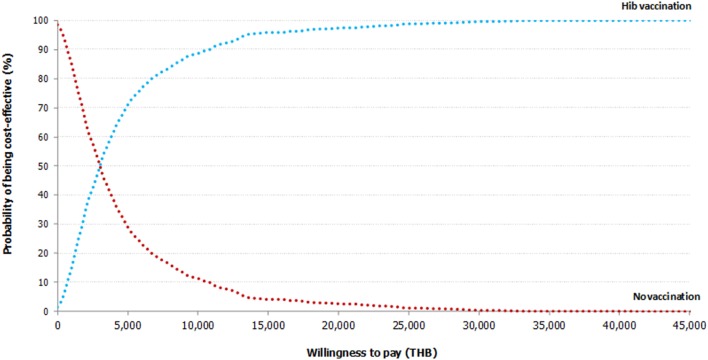
Cost-effectiveness acceptability curve. The cost-effectiveness acceptability curve shows the result of multivariate probabilistic sensitivity analysis based on 1,000 Monte Carlo simulations. If the willingness-to-pay threshold is set at THB 160,000 per QALY gained, the three-dose universal Hib vaccination program will have 100% probability of being cost-effective from societal perspective. THB, Thai baht; Hib, *Haemophilus influenzae* type b.

Multivariate probabilistic sensitivity analysis (PSA) was conducted to simultaneously examine the effects of all parameter uncertainty using a Monte Carlo simulation performed by Microsoft Excel 2003 (Microsoft Corp., Redmond, WA) (35). The various probability distributions were defined: (a) probability and utility ranging between zero and one would follow beta-distributions, (b) costs and length of stay normally positively skewed and positive would follow gamma-distributions, and (c) VE and relative risk of dying parameters would follow a log-normal distribution. 1,000 Monte Carlo simulations were run and results were presented as cost-effectiveness acceptability curve (Figure 3) (36). The expected net monetary benefit was calculated for WTP of NLEM 2012 threshold in Thailand in order to show the probability that DPT-HB-Hib vaccination program is cost-effective.

## Results

### Base-Case Analysis

Haemophilus influenzae type b vaccination program among 782,129 Thai children could avert 3,960 Hib-infected cases, which includes 240 Hib meningitis, 3,624 Hib pneumonia, and 96 Hib NPNM over 5 years (Table 4). It could prevent 27 hearing losses, 12 epilepsies, and 20 developmental deficits which are long-term Hib meningitis-related sequelae. The vaccination would avert 89 Hib-related deaths (13 due to Hib meningitis, 71 due to Hib pneumonia, and 5 due to Hib NPNM). Table 4 also presents incremental cost per capita of implementing both three-dose universal Hib vaccination program as THB 141.19 ($4.3). The incremental vaccination program cost of a three-dose vaccination schedule would require is THB 291.64 ($8.9) based on a unit cost of THB 148.5 ($4.5) per dose and wastage cost of 5% for DTP-HepB-Hib single-dose dosage form (39% for 10-dose vial), compared to THB 42.05 ($1.3) per dose of DTP-HepB multiple-dose preparation.

**Table 4:** Outcome measures from model-based estimation under base-case analysis.

Outcome measure	No haemophilus influenzae type b (Hib) vaccination	Hib vaccination	Difference
**Clinical outcomes (per 782,129 birth cohort)**			

Hib meningitis cases	251	10	−240
Hib pneumonia cases	3,742	118	−3,624
Hib non-pneumonia non-meningitis (NPNM) cases	99	3	−96

Total Hib-infected cases	4,092	132	−3,960

Hearing loss cases	28		−27
Epilepsy cases	13	1	−12
Developmental deficit cases	21	1	−20

Total Hib disability cases	61	3	−59

Hib meningitis deaths	25	1	−24
Hib pneumonia deaths	75	2	−72
Hib NPNM deaths	1	0	−1

Total Hib-infected deaths	101	3	−97

**Economic outcomes per capita: (Thai baht, year of costing 2014)**			

Direct medical care costs	122.21	295.61	173.40
Vaccination program cost	0.00	291.64	291.64
Treatment cost	122.21	3.97	−118.24
Direct non-medical care costs	33.31	1.11	−32.2
Total costs	155.52	296.71	141.19
Quality-adjusted life year (QALY) gained	33.58	33.71	0.13
Incremental cost per QALY		1,099.13	

*Base-case analysis in children aged under 5 years old whom preventive vaccine of Hib infection is indicated in societal perspective. Difference is calculated by values of Hib vaccination minus no Hib vaccination*.

Our model estimates that vaccinating children with Hib vaccine would save nearly THB 118.24 ($3.6) in direct medical care costs and THB 32.20 ($1) in direct non-medical care costs per vaccinated child. Therefore, a universal Hib infant vaccination with three-dose regimen is expected to lead to an ICER value of THB 1,099.13 ($33.5) per QALY gained under societal perspective.

### Sensitivity Analyses

In Figure 2, the one-way sensitivity analyses showed that the uncertainty around incidence of Hib pneumonia for those below 5 years of age led to the largest variation of ICER from THB 479 ($14.6) to THB 2,511 ($76.6) per QALY gained. All the remaining parameters seemed to have moderate to minimal impact on cost-effectiveness results. Results of the PSA based on 1,000 Monte Carlo simulations are presented in cost-effectiveness acceptability curve (Figure 3). Despite variation in base-case parameter inputs, around 1.40% of the simulated ICERs were in the lower-right hand quadrant, indicating that Hib vaccination program is always less costly and more effective than no vaccination (dominant). Another 98.6% of ratios which lie in the upper-right hand quadrant indicate that the vaccination program is more effective but also costlier than no vaccination. However, all simulated outcomes were well below the cost-effectiveness threshold line. As shown in Figure 3, the three-dose universal Hib vaccination program from societal perspective will have 100% probability of being cost-effective if the WTP threshold is set at THB 160,000 ($4,878) per QALY gained.

### Scenario Analyses

Based on current evidence of VE and coverage, 3 + 1 vaccination schedule provide costlier than 3 + 0 with the ICER was THB 1,835.53 ($56.5) per QALY gained. Two scenarios mentioned in the methodology section of higher cost estimates from previous costing study were also analyzed in order to provide an implication of varying Hib-infected direct medical care cost. For cost estimates using CPI for all cost items of previous costing study, the ICER was reduced to THB 56 ($1.7) per QALY gained, while it was cost-saving (less incremental cost with higher QALY gained) under separated labor cost adjustment using wage and salary with CPI adjustment for other cost items of previous costing study. In addition, when we used CFR from published article, which had higher CFR among Hib meningitis cases and lower CFR among Hib NPNM (Table 1), the ICER was slightly reduced to THB 1,098.23 ($33.5) per QALY gained. When analysis considered the effect of Hib vaccine only on Hib meningitis, ICER was increased to THB 31,753.21 ($968.1) per QALY gained.

### Threshold Analysis

At current pricing, universal Hib infant vaccination is unlikely to be cost-saving compared to no vaccination under societal perspective. The price of DTP-HepB-Hib vaccine should be THB ≤101 ($3.1) per dose for being cost-saving intervention. For cost estimates using CPI for all cost items of previous costing study, the maximum price per dose are THB 149 ($4.5) for cost-saving. In addition, prices should be THB 232 ($7.1) under separated labor cost adjustment using wage and salary with CPI adjustment for other cost items.

### Budget Impact Analysis

Based on a birth cohort of 782,129 children under base-case analysis, Thai government should invest about THB 228 ($7.0) million to implement 3 + 0 Hib vaccination program. However, total numbers of cases prevented can provide a cost reduction about THB 118 ($3.6) million. Therefore, an extra budget needed is about THB 110 ($3.4) million.

## Discussion

Haemophilus influenzae type b vaccination on children below five would substantially reduce the incidence of Hib-infected cases, leading to more than 100,000 QALYs gained. Thailand has had very low Hib infection which is believed to be a contributing factor to the former decision of not including Hib vaccination in EPI for Thai children (20, 25). However, our CUA incorporating most updated information showed that the preventive vaccine affirms a good value for money from societal perspective. This CUA study indicates that DTP-HepB-Hib vaccine offers a good value for money compared with existing EPI vaccine (DTP-HepB) at current market price. The ICER lies far below the Thailand’s standard ceiling threshold of THB 160,000 ($4,878) per QALY gained (16, 17). The break-even analysis also shows that the vaccination program would be cost-saving if the current vaccine price is reduced by one-thirds at THB 101 ($3.1) per dose where vaccination cost was offset by reduced treatment costs due to fewer Hib-infected cases. The budget impact analysis accounting for vaccine coverage and wastage under Thai context points to a reasonable incremental investment at current market price with routine Hib vaccine being included in EPI. The incremental investment is THB 141 ($4.3) for three doses compared to $8.9 (THB 292) per child vaccinated in Indonesia (37).

Haemophilus influenzae type b vaccine was found to be either cost-saving or cost-effective using country-specific cost-effectiveness threshold in most of the other countries such as Indonesia, India, Belarus, and Uzbekistan (37–40). In the same region, two CUA studies in Indonesia found that Hib vaccine is a highly cost-effective intervention. The confirmed Hib meningitis incidence used in both studies was low and was from prospective study in the selected catchment area as representative of all Indonesia, like we used the Thai prospective incidence study in our analysis (20, 41). However, one CBA study published in South Korea presented non-cost-effective results similar to Thai study, main factors for showing non-cost-effectiveness discussed in the two CBA studies were vaccine price, which was quite high at the time of analysis and the low incidence of Hib-related infection in both South Korea and Thailand (14, 42).

Our study did not incorporate herd immunity into the model because of a paucity of information related to herd immunity of Hib vaccine. This assumption is in line with previous cost-effectiveness analyses, which also did not incorporate herd immunity into the model. Since our findings showed that Hib vaccine was cost-effective, inclusion of herd effect would make it even more favorable. Therefore, this lack of herd effect incorporation will not change the overall conclusion of our study.

Even though our findings were sensitive to Hib pneumonia incidence, however it did not reverse our conclusion. It is worth noting that Hib pneumonia incidence was assumed to be 4–10 times of that of meningitis same as previous CBA study in Thailand (14). We performed a more conservative analysis considering only the effect of Hib vaccine on Hib meningitis and found that ICER was still below the cost-effectiveness threshold. This reflects the robustness of our findings. Furthermore, the results in our analysis were most sensitive to Hib disease incidence, vaccine price, and discount rate. This is in line with the report from the systematic review of Hib vaccine economic evaluations which indicated that previous studies were also most sensitive to these three parameters (43). In addition, the PSA results of all 1,000 iterations fall below the national cost-effectiveness threshold.

Input parameters used in this model were Thailand-specific and obtained from high-quality sources, including systematic reviews and meta-analyses. We used Hib incidences from local prospective population-based study to estimate number of infected cases (20, 25). VE was obtained from a meta-analysis (15). Contrary to most of the vaccine CEA studies that used vaccine efficacy data for projection, the use of VE is more realistic because it was obtained from real-world evidence. Furthermore, the use of domestic inputs in the model makes health authorities better informed in introducing Hib vaccine into EPI.

This study has some limitations. First, this study adopted a static model rather than transmission dynamic. The static model used in the current analysis is unable to estimate herd effect. Thus, the current results should be deemed conservative. Using dynamic transmission model would expect to generate more favorable outcomes. Second, we used Hib infection incidence from five provinces to estimate cases for the whole country. Although all included provinces are representative of all regions of the country; however, we do not exactly know whether this figure is an over- or under-estimate of the true incidence at national level. This uncertainty was addressed in 1-way sensitivity analysis and it showed that Hib pneumonia incidence is the most influential parameter. However, it still showed cost-effectiveness when the Hib pneumonia incidence was not considered in scenario analysis. Finally, this model used one year as the model’s cycle length. This limits the recurrence of Hib infection within one year. However, the cycle length was agreed by our stakeholders, which included clinicians, researchers, and health payers. The cycle length is also similar to previous cost-effectiveness analyses of Hib vaccine.

There has been one economic evaluation of universal Hib vaccination program in Thailand using CBA approach where tangible and intangible costs and benefits were captured (14). They measured and valued intangible costs and benefits by surveying the WTP among parents who want their children to get Hib vaccine. It was shown that Hib vaccination program produced negative net benefit thus was not cost-effective compared to no vaccination program without including intangible costs. However, the intangible cost itself is difficult to quantify in monetary unit and is dependent on eliciting approach used (44). In addition to this uncertainty of CBA, HTA guideline of Thailand recommended CUA as a national preferred approach for economic evaluation. Therefore, this study was performed in line with current HTA guideline and our results are thus useful to enable comparison with CUAs of other health interventions as effectiveness is standardized as QALYs. This also allows the allocative efficiency among competed health measures under a limited health care budget. It is also important to note that the cost-effectiveness ceiling threshold used in our analysis is based on the local preference of decision makers/bodies (16, 17). Under other circumstances, they may have their own preference regarding health investment. Therefore, it is encouraged that audiences compare the results to any threshold considered as appropriate. As aforementioned, Thailand is in the minority among many countries that have already implemented Hib vaccine for their citizens. The main reason for this vaccination program not being adopted in Thailand is due to low Hib-infected incidence. However, our results using the updated information show that this vaccination program would be cost-effective even with low disease burden. Our analysis showed reconsidering of Hib vaccine to be included in EPI may be warrant based on these economic evaluation results along with other important aspects as well. In summary, DTP-HepB-Hib vaccination program for Thai children would be a very cost-effective intervention and is likely to produce a budget impact affordable to government. Furthermore, its delivery schedule is also aligned with the implemented DTP-HepB vaccine. The inclusion of DTP-HepB-Hib in Thailand’s EPI is highly encouraged.

## Author Contributions

SK and NC contributed in the study conception, model conceptualization, critical review of input parameters, model analyses and interpretation, consultation-meeting arrangement, and manuscript writing. CM and ST participated in study conception, reviewed the model outputs from the clinical viewpoint and consultation-meeting arrangement, and participated in manuscript drafting. PD and DW participated in critical review of input parameters, model analyses and interpretation, and manuscript writing. All authors reviewed the final draft and collectively decided to submit for publication.

## Conflict of Interest Statement

CM and ST are employees of the NVI—a not-for-profit organization. The rest of authors had funding support from NVI for conducting the study and had no other relationships or activities that could appear to have influenced the submitted work.
