# Supplementary material for: Economic Evaluation and Budget Impact Analysis of Vaccination against Haemophilus influenzae Type b Infection in Thailand
Source: Front Public Health. 2017 Nov 20;5:289. doi: 10.3389/fpubh.2017.00289 (PMC5701919; doi:10.3389/fpubh.2017.00289)
Supplement: Supplementary file 1 [file data_sheet_1.docx]

Supplementary Material

**Economic evaluation and budget impact analysis of vaccination against *Haemophilus influenzae* type b infection in Thailand**

Surachai Kotirum, PharmD^1, 2^, Charung Muangchana, MD, PhD^3,†^, Sirirat Techathawat, MSc^3,†^, Piyameth Dilokthornsakul, PharmD, PhD^4^, David Bin-Chia Wu, PhD^1^, Nathorn Chaiyakunapruk, PharmD, PhD^1,4, 5 *^

^1^School of Pharmacy, Monash University Malaysia, Bandar Sunway, 47500, Malaysia

^2^Social and Administrative Pharmacy Department, Faculty of Pharmacy, Rangsit University, Muang, Pathumthani, Thailand

^3^National Vaccine Institute (Public organization), Ministry of Public Health, Nonthaburi, 11000, Thailand

^4^Center of Pharmaceutical Outcomes Research (CPOR), Department of Pharmacy Practice,

Faculty of Pharmaceutical Sciences, Naresuan University, Phitsanulok, 65000, Thailand

^5^School of Pharmacy, University of Wisconsin, Madison, WI 53706, USA

^*^ Correspondence and requests for materials should be addressed to N.C. (email: [nathorn.chaiyakunapruk@monash.edu](mailto:nathorn.chaiyakunapruk@monash.edu))

^†^ See Author’s contribution on the last page

**(Figure 1.)** Model used for evaluating costs and health-related outcomes of DTP-HepB-Hib vaccination national program compared to no vaccination (The structure of the vaccination program node is same to no vaccination node).

###

**(Figure 2.)** Tornado diagram of base-case analysis (3 + 0 vaccination schedule with NHSO costing data).

**(Figure 3.)** Cost-effectiveness acceptability curve
